# Histopathological features of condylar hyperplasia and condylar Osteochondroma: a comparison study

**DOI:** 10.1186/s13023-019-1272-5

**Published:** 2019-12-16

**Authors:** Jingshuang Yu, Tong Yang, Jiewen Dai, Xudong Wang

**Affiliations:** 1 Department of Oral and Craniomaxillofacial Surgery, Ninth People’s Hospital Shanghai Jiao Tong University School of Medicine, No.639 Zhizaoju Road, Huangpu District, Shanghai, 20011 People’s Republic of China; 2 Shanghai LinkedCare Information Technology Co., Ltd, Shanghai, People’s Republic of China

**Keywords:** Mandibular condylar hyperplasia, Condylar osteochondroma, Histopathology, PCNA, EXT1

## Abstract

**Background:**

Both mandibular condylar hyperplasia and condylar osteochondroma can lead to maxillofacial skeletal asymmetry and malocclusion, although they exhibit different biological behavior. This study attempted to compare the histological features of mandibular condylar hyperplasia and condylar osteochondroma using hematoxylin-and-eosin (H&E) staining, and immunohistochemistry staining of PCNA and EXT1 with quantitative analysis method.

**Results:**

The H&E staining showed that condylar hyperplasia and condylar osteochondroma could be divided into four histological types and exhibited features of different endochondral ossification stages. There was evidence of a thicker cartilage cap in condylar osteochondroma as compared condylar hyperplasia (*P* = 0.018). The percentage of bone formation in condylar osteochondroma was larger than was found in condylar hyperplasia (*P* = 0.04). Immunohistochemical staining showed that PCNA was mainly located in the undifferentiated mesenchymal layer and the hypertrophic cartilage layer, and there were more PCNA positive cells in the condylar osteochondroma (*P* = 0.007). EXT1 was mainly expressed in the cartilage layer, and there was also a higher positive rate of EXT1 in condylar osteochondroma (*P* = 0.0366). The thicker cartilage cap, higher bone formation rate and higher PCNA positive rate indicated a higher rate of proliferative activity in condylar osteochondroma. The more significant positive rate of EXT1 in condylar osteochondroma implied differential biological characteristic as compared to condylar hyperplasia.

**Conclusions:**

These features might be useful in histopathologically distinguishing condylar hyperplasia and osteochondroma.

## Background

Osteochondroma is described as osteocartilaginous exostosis [1]. It is considered the most common tumor of skeletal bones, comprising approximately 35 to 50% of all benign bone tumors [2], but it is rarely found in the jaw [3]. Condylar hyperplasia is characterized by a unilateral non-neoplastic overgrowth of the condyle and the mandible [4]. Condylar hyperplasia is a self-limiting disease that is generally observed as growth in young patients between the ages of 11 and 30 years [5]. Both mandible condylar hyperplasia and condylar osteochondroma can lead to severe maxillofacial skeletal asymmetry and malocclusion. The low condylectomy has been known to stop the continuous deviation [6, 7]. Whereas osteochondroma is defined as a benign tumor, it means that there is differential biological behavior between these two diseases and results in different treatment strategies. Moreover, malignant transformation to chondrosarcoma and multiple hereditary osteochondromatosis is rare but was observed in osteochondroma [8, 9]. Therefore, differential diagnosis of these two diseases is necessary.

Now differential diagnosis of these two mandibular diseases tends to depend on non-invasive diagnostic examination, including X-ray, CT and MRI. However, these methods present with inherent limitations, and the cell behavior based on pathological information is still considered the definitive choice for diagnosis. Furthermore, studies of the pathogenesis and molecular biology of mandible condylar hyperplasia and condylar osteochondroma currently remain at an initial stage of investigation, and the qualitative H&E staining results showed no characterized cell behavior between these two diseases [10].

Both diseases are characterized by excessive growth and enlargement of the mandibular condyle. Therefore, cell proliferation is a key evaluation marker, and a quantitative and specific staining method is necessary to effectively make a differential diagnosis of mandible condylar hyperplasia and condylar osteochondroma. Besides, PCNA (Proliferating Cell Nuclear Antigen) is a nuclear protein that is expressed in the G1-M phases of the cell cycle, but is maximally expressed in the late G1-S phase [11]. PCNA is involved in DNA replication, repair, cell cycle regulation, apoptosis and other important cellular events [12]. Thus, changes in the expression levels of PCNA are closely related to DNA synthesis and play a key role in the initiation of cell proliferation, which can be a good clinical indicator that reflects the state of cell proliferation. In this study, immunohistochemical staining of PCNA was used to observe the proliferative activity status for both diseases.

While the majority of osteochondromas present as solitary (i.e., non-hereditary) lesions [13, 14], approximately 15% of osteochondromas occur as multiple osteochondromas (MO), an autosomal dominantly inherited disorder, which was previously referred to as hereditary multiple exostoses [9, 15]. The EXT1 and EXT2 genes have been identified for MO [16], especially in the context of the loss of the remaining EXT1 wild type allele that was demonstrated in hereditary osteochondromas [17]. Regarding solitary osteochondromas, EXT1 homozygous deletions are found to be confined to the cartilaginous cap in sporadic cases [18], confirming that EXT1 is required for osteochondroma development. Therefore, we intended to detect EXT1 expression in condylar osteochondroma and condylar hyperplasia to preliminarily explore the pathogenesis of condylar osteochondroma and condylar hyperplasia.

In this current study, we attempted to quantitatively describe the histological and molecular features of mandibular condylar hyperplasia and condylar osteochondroma. Subsequently, the histological and molecular difference between both diseases was also described and discussed.

## Results

### Patient information

**Fig. 1:**
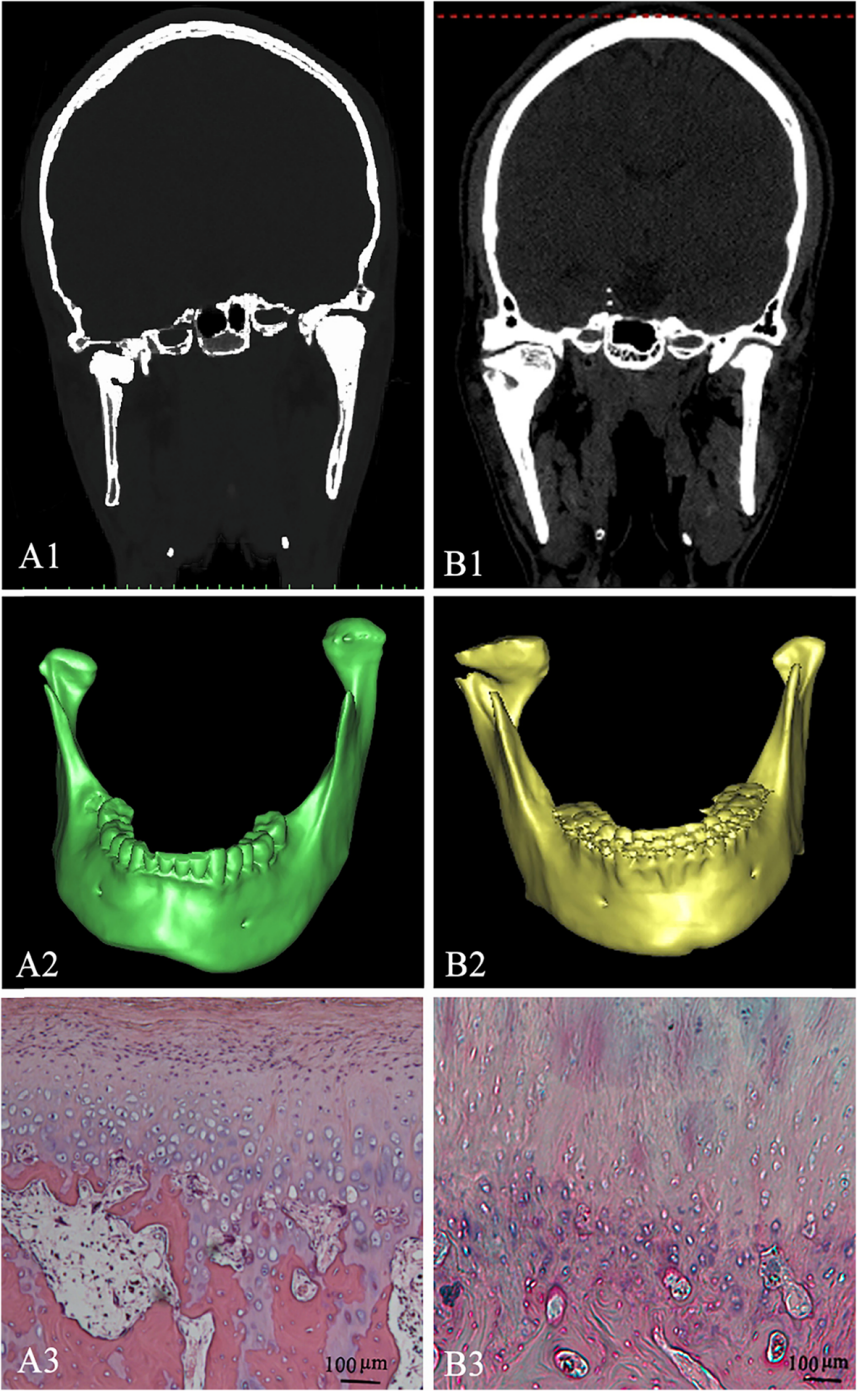
**a1**-**a3**. Coronal view,3D CT and H.E. stained of the lesion in a condylar hyperplasia of a 21-year-old patient. **b1**-**b3**. Coronal view,3D CT and H.E. stained of the lesion in a condylar osteochondroma of a 22-year-old patient

The diagnosis of condylar osteochondroma and condylar hyperplasia were made by experienced maxillocraniofacial surgeons, radiologists and pathologists, and diagnoses were based on the clinical symptoms, CT scanning characteristics and H&E staining (Fig. 1). Thus, the 33 patients (18 condylar osteochondroma and 15 condylar hyperplasia) were then divided into four types (Table 1, and Table 2). There were more female patients with left side priority in both condylar hyperplasia and condylar osteochondroma as compared male cases. The mean age of the patients in the condylar hyperplasia group was 26 ± 4.8 years of age, and the mean age in the condylar osteochondroma group was 32 ± 10.2 years of age. The Satterthwaite method T-test result showed that condylar osteochondroma patients exhibited a senior age as compared patients in the condylar hyperplasia group (*P = 0.448* < 0.05).7

**Table 1:** Patients’ Information in Condylar Hyperplasia Group

Classification of Condylar Hyperplasia	Number	Mean Operation Age	Gender	L/R
Type I	4	27	3 M, 1 F	3 L,1 R
Type II	1	29	/ M, 1 F	/ L,1 R
Type III	6	24	4 M, 2 F	3 L, 3 R
Type IV	4	29	1 M, 3 F	3 L, 1 R
Total	15	26	9 M, 6 F	9 L, 6 R

*M* Male, *F* Female

**Table 2:** Patients’ Information in Condylar Osteochondroma Group

Classification of Condylar Osteochondroma	Number	Mean Operation Age	Gender	L/R
Type I	3	41	2 M, 1 F	3 L, / R
Type II	8	25	4 M, 4 F	2 L, 5 R
Type III	7	35	5 M, 2 F	6 L, 2 R
Type IV	/	/	/	/
Total	18	32	11 M, 7 F	11 L, 7 R

*M* Male, *F* Female

### H&E staining

**Fig. 2:**
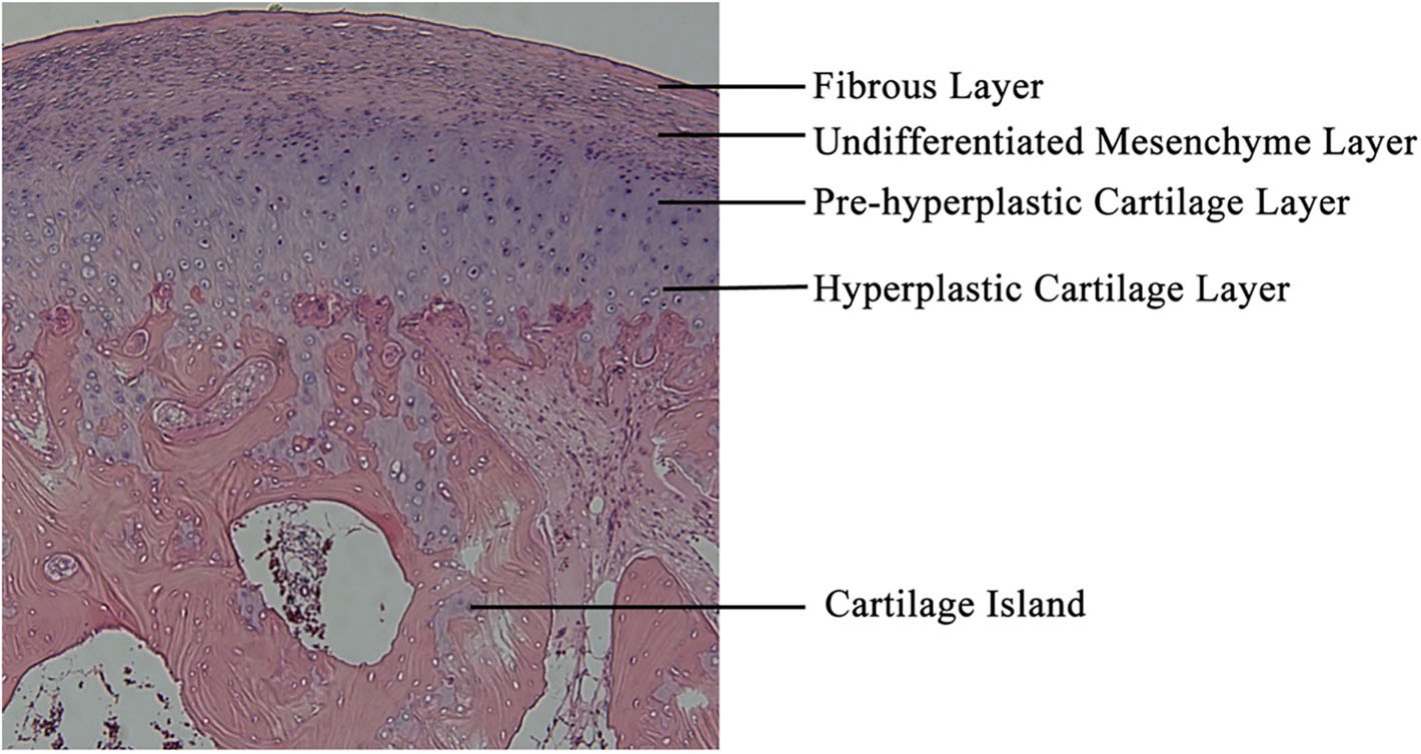
The H.E. staining of condylar osteochondroma. The fibrous layer, the undifferentiated mesenchyme layer, the pre-hyperplastic and hyperplastic cartilage layer are shown in the H.E. staining of a 21-year-old patient condylar osteochondroma, and cartilage islands are scattered throughout the underlying trabecular bone. (H.E., ×50)

**Fig. 3:**
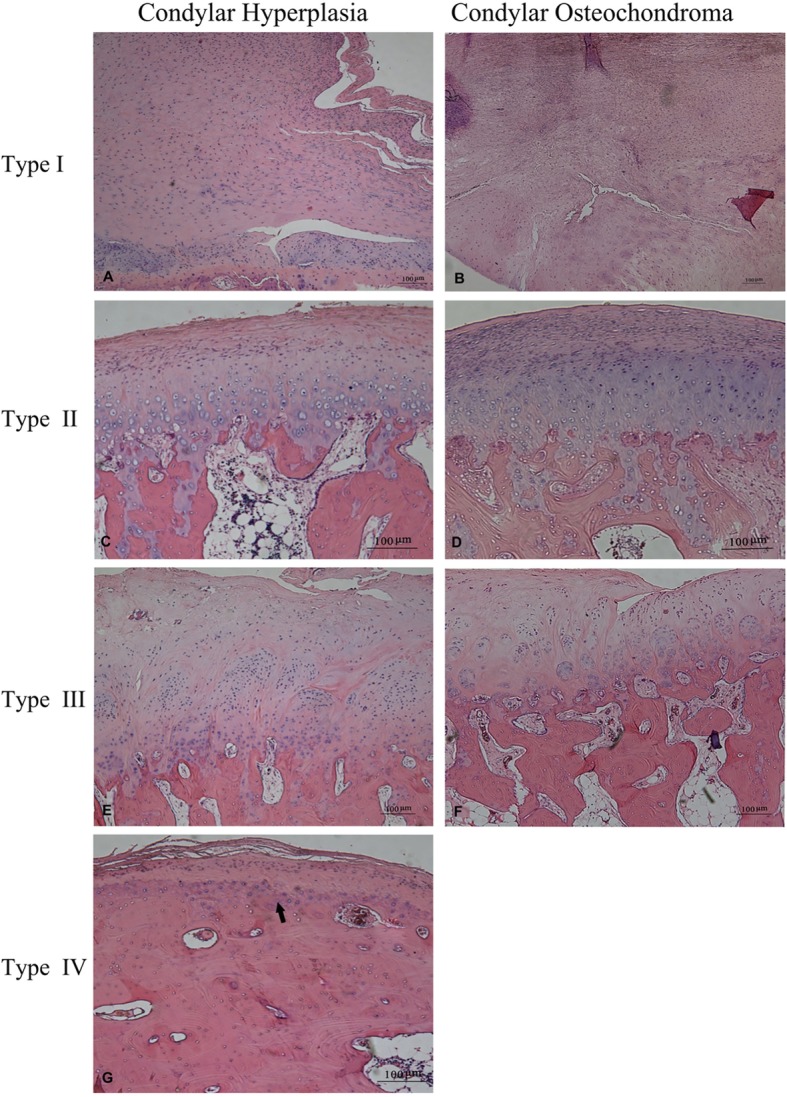
Type I. **a**.24-year-old, female, condylar hyperplasia. **b**.55-year-old, male, condylar osteochondroma (H.E.×50). Type II. **c***.*21-year-old, male, condylar hyperplasia. **d***.*21-year-old, male, condylar osteochondroma (H.E. × 50). Type III. **e***.*29-year-old, female, condylar hyperplasia. **f***.*21-year-old, male, condylar osteochondroma (H.E. × 50). Type IV. **g***.*35-year-old, male, condylar hyperplasia. Tidemark (Arrow) appears as a basophilic wavy line at the interface between the calcified and hypertrophic layer of the condylar cartilage (H.E.×50)

Both condylar hyperplasia and condylar osteochondroma showed a cartilage cap that covered the surface of the condyle. The cartilage cap was divided into four layers: the fibrous layer, undifferentiated mesenchyme layer, cartilage layer including pre-hypertrophic and hypertrophic chondrocytes and the calcified cartilage layer (Fig. 2). The condylar cartilage exhibited features of different endochondral ossification stages and was divided into four histological types based on their H&E staining features:

(1) Type I (Fig. 3a and b): The fibrous layer was continuous, and undifferentiated mesenchymal layers in the cartilage cap were very thick. The number of spindle-shaped or elliptic small cells was both large and dense. The underlining pre-hypertrophic chondrocyte layer, with a few hypertrophic and vacuolar chondrocytes, was thinner than the undifferentiated mesenchymal layer. The cartilage structure was continuous with the underlying bone, and the condylar bone surface showed intermittent absorption. There was an almost complete absence of a cartilage island in the inferior cancellous bone, and the bone under the cartilage displayed a patchy distribution.

(2) Type II (Fig. 3C and D): The structure of cartilage cap was clear. But the fibrous and undifferentiated mesenchymal layer was not as thick as was seen for type I. The underlining pre-hypertrophic and hypertrophic chondrocyte layer was getting thicker. The fusion and absorption area in the connecting part with the inferior bone became larger, and the number of cartilage islands was increased in the inferior cancellous bone.

(3) Type III (Fig. 3e and f): The structure of the cartilage cap was also clear, and the undifferentiated mesenchymal layer, pre-hypertrophic chondrocyte layer and hypertrophic chondrocyte layer almost exhibited similar thicknesses. Chondrocytes were located along the condylar growth direction, and secretion of the cartilage matrix was increased with obvious basophilic blue staining in the interstitial area. The cartilage cap and condylar bone were fused and continuous, and there were more cartilage islands among the cancellous bone than were found for type II.

(4) Type IV (Fig. 3g): The undifferentiated mesenchymal cell layer that was found below the fibrous layer was thinner, and there was no obvious cartilage layer. In some areas, the fibrous layer was directly connected with the underlying bone with a thin layer of basophilic bone response line, called “Tidal lines”, appearing on the condyle bone surface. The bone cancellous structure directly connected with cartilage was thinner, where cartilage islands was rare. In addition, in our study, type IV could only be found in condylar hyperplasia cases.

**Fig. 4:**
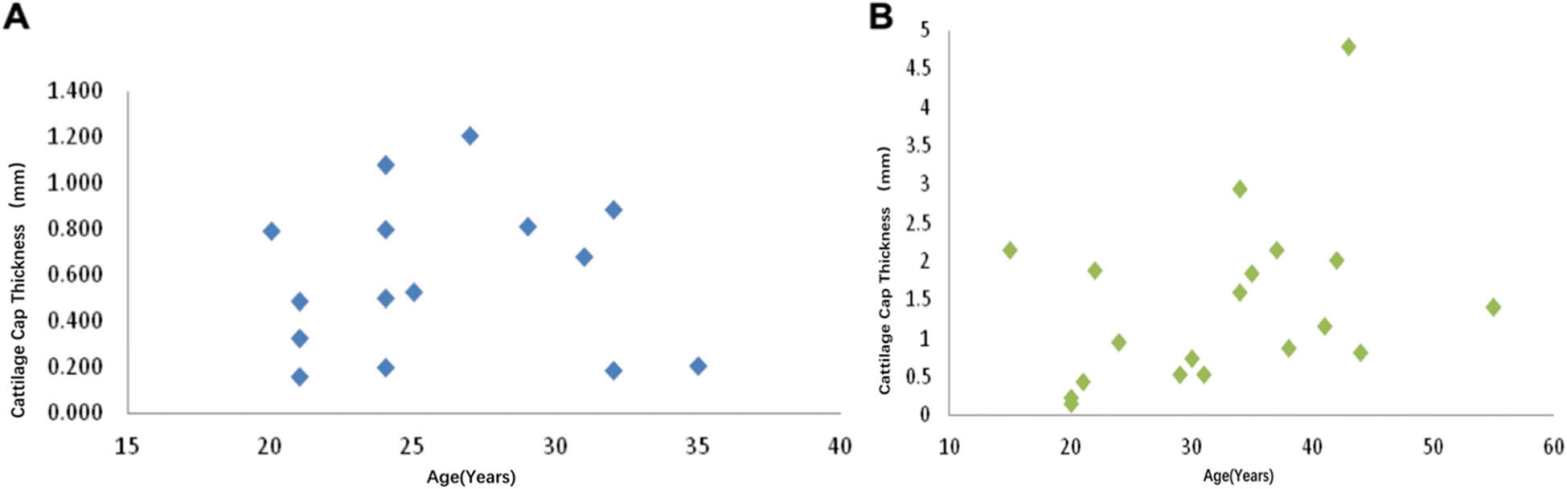
**a**. Relationship between age and thickness of cartilage cap of cases of mandibular condylar hyperplasia. **b**. Relationship between age and thickness of cartilage cap of cases of mandibular condylar osteochondroma

The thickness of the cartilage cap was combined with the undifferentiated mesenchymal layer and the cartilage layer. There was no linear relationship between the thickness of the cartilage cap and age when based on the scatter gram in both the condylar hyperplasia group (r = 0.00255, *p* = 0.9928) and the condylar osteochondroma group (r = 0.33409, *p* = 0.1620) (Fig. 4a and b).

There was a statistically significant thicker cartilage cap (*p* = 0.01, *p* < 0.05) and the chondrocyte layer (*p* = 0.015, p < 0.05) in condylar osteochondroma when compared with condylar hyperplasia. The percentage of bone formation in condylar osteochondroma was larger than found in condylar hyperplasia (*p* = 0.04, *p* < 0.5). Whereas the thickness of the fibrous layer, undifferentiated mesenchymal cell layer, the number of cartilage islands and the depth of infiltration of the cartilage islands were not significantly different between groups (Table 3).

**Table 3:** Histological Measurement Results

Groups	Condylar Hyperplasia	Condylar Osteochondroma	Wilcoxon Test
Data	X ± SD	X ± SD	*P* Value
Thickness of Fibrous Layer (mm)	0.105 ± 0.100	0.115 ± 1.135	0.86
Thickness of Undifferentiated Layer (mm)	0.371 ± 0.327	0.796 ± 0.826	0.32
Thickness of Cartilage Layer (mm)	0.221 ± 0.136	0.721 ± 0.900	0.015
Thickness of Undifferentiated Layer + Cartilage Layer (mm)	0.592 ± 0.337	1.438 ± 1.119	0.01
Thickness of Cartilage Cap (mm)	0.690 ± 0.376	1.581 ± 1.191	0.018
Number of Cartilage Island	5.267 ± 5.133	9.333 ± 8.534	0.28
Depth of Cartilage Island Infiltration (mm)	1.596 ± 1.851	1.786 ± 2.482	1.00
Area of Bone Formation (%)	47.362 ± 13.060	57.542 ± 12.284	0.04
PCNA (%)	11.932 ± 9.593	19.097 ± 9.528	0.007

### Immunohistochemistry staining

**Fig. 5:**
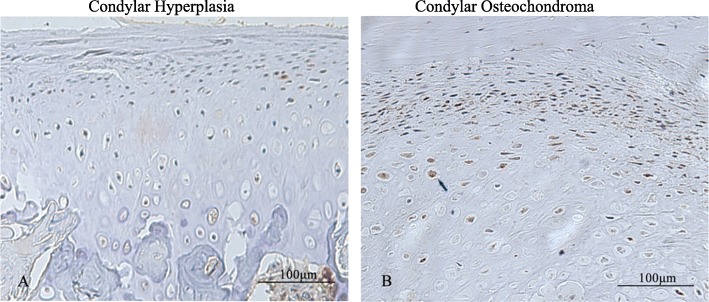
Immunohistochemical staining of PCNA. **a***.* Condylar hyperplasia. **b***.* Condylar osteochondroma. PCNA dots (arrow) scattered in large numbers in the nucleus of the cells

The immunohistochemistry staining results showed that PCNA was mainly located in the undifferentiated mesenchymal layer and pre-hypertrophic and hypertrophic cartilage layer (Fig. 5c), mainly in the pre-hypertrophic cell. In addition, there were obviously more PCNA positive cells in condylar osteochondroma (*p* = 0.007, Table 3, Fig. 5a and b).

**Fig. 6:**
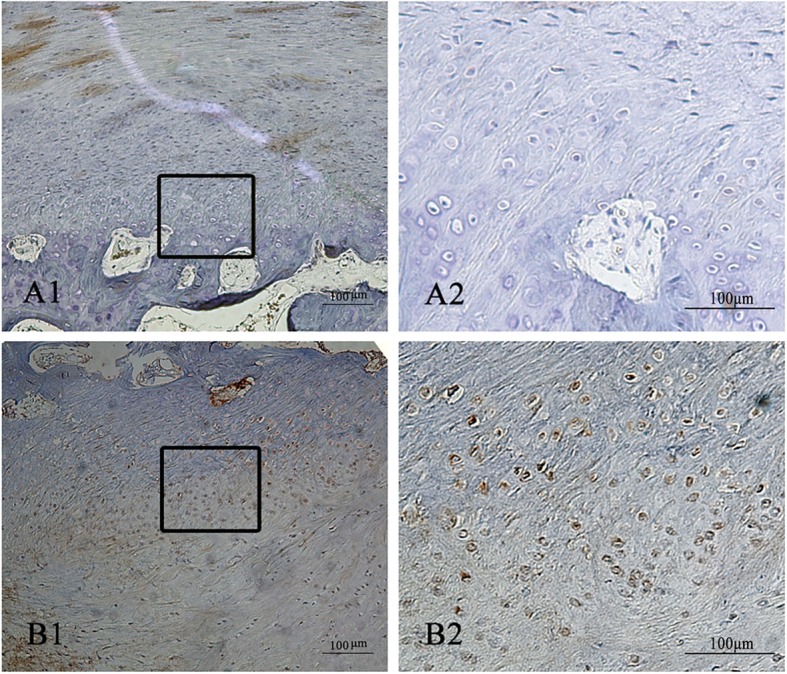
Immunohistochemical staining of EXT1. **a***.* Condylar hyperplasia (Type I) (A1x20, A2× 200). **b***.* Condylar Osteochondroma (Type II) (B1x20, B2 × 200)

**Fig. 7:**
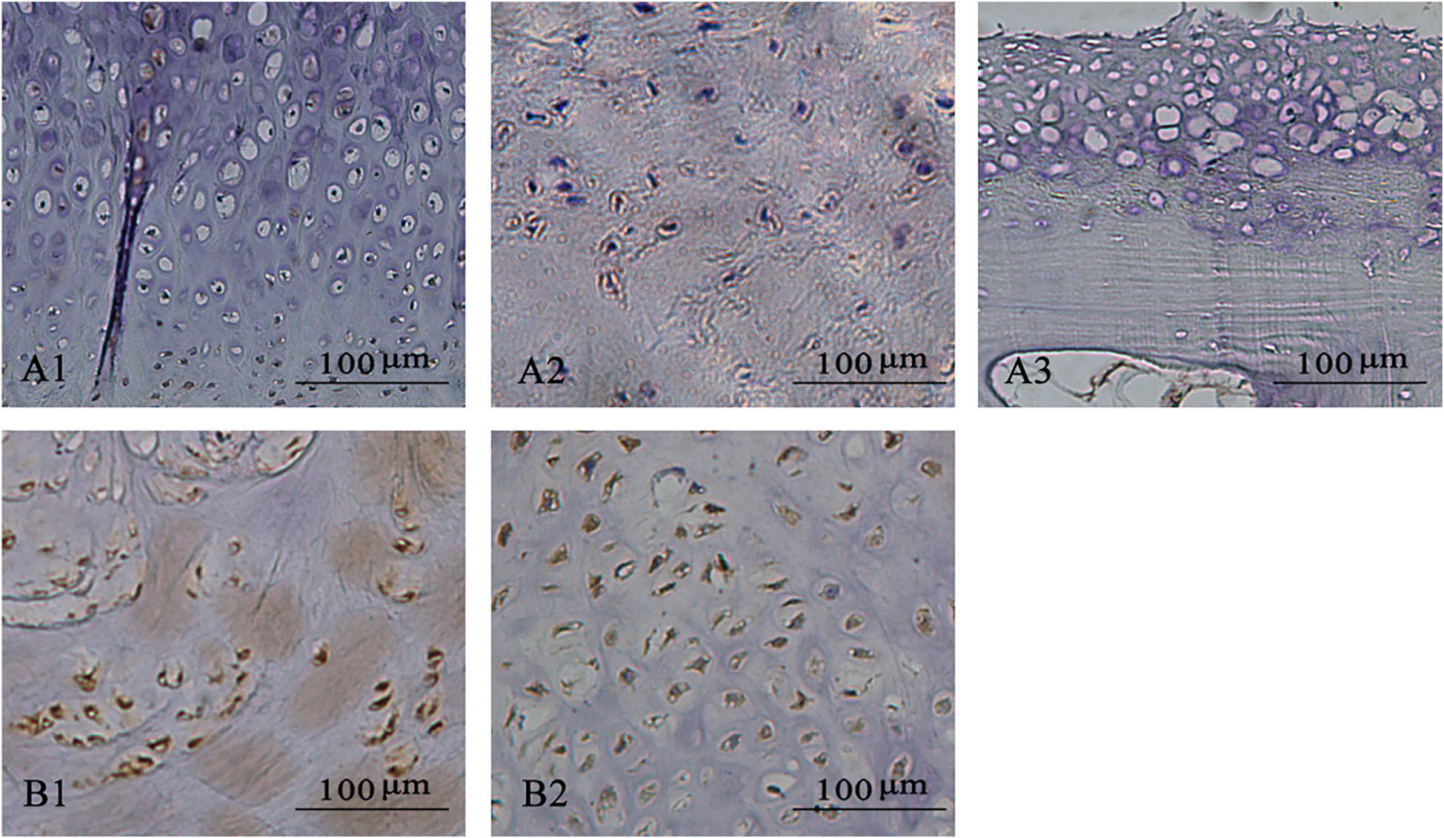
Immunohistochemical staining of EXT1 of different Phases of lesions. **a***.* Condylar hyperplasia, A1 Type II, A2 Type II, A3 Type IV. **b***.* Condylar Osteochondroma, B1 Type II, B2 Type III

EXT1 was mainly expressed in the cartilage layer (Fig. 6), and there was a higher positive rate of EXT1 in the condylar osteochondroma group (*p* = 0.0366, *p* < 0.5, Table 4 and Fig. 7).

**Table 4:** EXT1 Positive or Negative Patients in Mandibular Condylar Hyperplasia and Condylar Osteochondroma

Group	Condylar Hyperplasia		Total	Condylar Osteochondroma		Total
	EXT1(+)	EXT1(−)		EXT1(+)	EXT1(−)	
Type I	0	4	4	2	1	3
Type II	1	0	1	4	3	7
Type III	3	3	6	6	2	8
Type IV	0	4	4	/	/	/
Total	4	11	15	12	6	18

The thicker cartilage cap, the larger bone formation rate and the higher PCNA positive rate indicated a higher proliferative activity of condylar osteochondroma. The higher EXT1 positive rate in condylar osteochondroma implied different biological characteristics as compared to condylar hyperplasia. These features might be useful in histopathologically distinguishing condylar hyperplasia and osteochondroma.

## Discussion

Precisely how to differentiate condyle osteochondroma from condylar hyperplasia remains controversial. Not only the clinical manifestations, but also histological description of these two diseases present similar aspects [10]. The different classifications for condylar hyperplasia or osteochondroma by researchers have been developed in order to standardize the concept of the diseases and treatment [19–22]. The current basis for diagnosis and treatment was comprehensive sequence including the clinical examination of facial outcome and dental analyses, radiographic features for the analysis of the condyles, SPECT and histological examination for both condylar hyperplasia and condylar osteochondroma. However, the cellularity of the disease, the essential and directive evidence to define the disease, is still to be acquired by histological analysis. In our study, the quantitative histological analysis was carried out based on 15 cases condylar hyperplasia and 18 osteochondroma according to our hospital’s diagnosis.

It was reported that cartilaginous tumors are nearly exclusively found in bones arising from endochondral ossification, and different cartilaginous tumors represent different stages of chondrogenesis [23]. The pathology of these cartilaginous tumor tissue exhibited three layers: (1) the surface fibrous connective tissue. (2) the middle layer with cap-like cartilaginous tissues and matrix. (3) mature trabecular bone beneath the cartilaginous layer. The morphology was in agreement with the process of endochondral ossification [24, 25]. In our H&E staining study, similar structures were observed in both the condylar hyperplasia and condyle osteochondroma groups. In addition, four layers can be separated for a condylar that is formed by the layered chondrocytes (Fig. 2), which is different from chondrocytes found in the growth plate. Ji et.al clearly observed the layered structure of condylar cartilage named “hierarchical structure” by using safranin O staining and safranin-fast green staining [26]. The “hierarchical structure” was also observed and described in the condylar hyperplasia [27].

In Slootweg and Muller’s study [20], they divided condylar hyperplasia into 4 types based on the infiltration of islands of cartilaginous tissue and the size of the fibrocartilage layer: 1) a type I condyle, which was characterized by the presence of hyaline growth cartilage, whereas the type II condyle exhibits as fibrocartilage. The histological architecture of type III was greatly distorted, with irregular fields of hyaline cartilage that merge with the underlying spongy bone. The type IV condyle with features of cell-poor fibrous cartilage tissue, showed a “burned-out appearance”. In this classification,only use the islands of cartilaginous island as the parameter was lack of patterns of normality and the absence of analytical patterns of the pathological structure [10]. Chondrocytes in osteochondroma go through similar progressive differentiation, including resting, proliferating, pre-hypertrophic and hypertrophic stages, and eventually undergoing programmed cell death, providing the scaffolding on which new bone is formed. Moreover, adjacent to the region in which chondrocytes undergo apoptosis, blood vessels continue to attract new osteoblasts to lengthen the bone [28, 29]. Based on the work above, the structure of cartilage accords with different stages of endochondral ossification process was used to renew the four types. Type I-III was slightly different to that proposed by Slootweg, exhibiting endochondral ossification process in both the condylar hyperplasia and condylar osteochondroma group.

For type IV, the special tidemark-like structure was observed only in hyperplasia group. The tidemark reflects the metabolism of the cartilage area and that below the calcified area. The immature bone tissue exhibits more matrix than its mature one. Thus, immature bone tissue tends to stain blue by H&E staining, and mature bone tissue tends to dye red in response to eosin staining. In 1953, Fawns [30] observed unique dyeing lines, which were defined as a “Tidemark,” that were located between articular and calcified cartilage, which was not observed in bone tissues of the developing animal. Chen et al., [54] reported that in normal condylar, tidemark was only observed in mature condyle process. When condylar activity burns out, the tidemark appears. In this current study, type IV only appeared in condylar hyperplasia, and the maximum age for surgery in condylar hyperplasia in our department was 35 years,senior than previous reports of 11–30 years [20, 31, 32],which indicated that condylar hyperplasia might be a self-limited developmental disease but condyle proliferation can be active at all ages without upper limit. One reason for that can be the different clinic time of the patients, but it needs more cases and longer following up periods. It was also reported that osteochondroma in long bones exhibited self-limited characteristics when the growth plate was closed and it ceased growing [23]. However, there is no related study proving that the mandible condyle osteochondroma is similarly defined by self-limited characteristics. In our study, we did not find a Tidemark in type IV. There are two possible mechanisms: one posits that condylar osteochondroma did not display a self-limited feature, and on the other hand, the other one might have been due to faster proliferation of condylar osteochondroma that resulted in facial asymmetry. Thus, when a patient visits the physician, the disease presents still in the dynamic phase of proliferation. Our study also showed that the mean age of patients in the condylar osteochondroma group was 32 ± 10.2 years of age and 26 ± 4.8 years of age in hyperplasia group, which can be inferred that older facial asymmetry patients might suffer from condylar osteochondroma. It was suggested that special attention should be paid to the possibility of condylar osteochondroma in patients over 30 years old who seek treatment for facial asymmetry, especially for mandibular deformity.

The analysis of thickness of cartilage cap, the fibrous layer, the undifferentiated mesenchymal and the cartilage cell layer, the depth of average infiltration, the numbers of cartilage islands and the PCNA positive rate were attempted to compare the proliferation of two diseases. The mean of the total cartilage cap in condylar osteochondroma was thicker than was found in condylar hyperplasia. Considering surgery may damage condylar organization [33], especially the condylar surface fibrous layer, we thus summed up the undifferentiated mesenchymal cell layer and chondrocyte layer and found that the condylar osteochondroma group remained thicker than that found in the hyperplasia layer. The unmineralized cartilage that scatters in the trabecular bone under the cartilage cap was not a sign of a malignant lesion. However, it reflects the speed of endochondral ossification. Under conditions where there is an increased appearance of a cartilage island and infiltration depth, the faster the condylar grows [20]. Indeed, Gray [32] reported that the density of the cartilage island was positively correlated with the infiltration depth, which is directly associated with the degree of condylar hyperplasia. However, there are also some opposing conclusions. Slootweg and Muller [20] reported no direct relationship with the above index. In addition, Eslami’s research showed no significant difference between condylar hyperplasia and the normal condyle [34]. In our study, the number of cartilage islands and the depth of infiltration in condylar osteochondroma exceeded that seen in the condylar hyperplasia group without any significant difference. However, bone formation area in condylar osteochondroma exceeded that seen in condylar hyperplasia and was significantly different. Moreover, the positive rate of PCNA staining in condylar osteochondroma was obviously higher than the rate found in condylar hyperplasia. Taking the above into account, our study implied that condylar osteochondroma exhibited a higher proliferative activity than condylar hyperplasia, without any evidence of condyle activity burn out. However, whether the number of cartilage islands and the depth of infiltration represent an index of condyle proliferation needs further study.

Apart from the cell proliferation conditions, in the previous study, it was clearly shown that osteochondromas morphologically resemble the normal growth plate, arising from endochondral ossification. In addition, in our pathological analysis, both condylar osteochondroma and condylar hyperplasia represent features of endochondral ossification. However, osteochondroma is still defined as a real tumor. It was demonstrated by cytogenetic abnormalities, aneuploidy and loss of heterozygosity (LOH) found in the cartilaginous cap, which also involved the EXT gene location. Additionally, the loss of function or mutation of EXT1 is crucial in the pathogenesis of solitary as well as hereditary osteochondromas [35]. The EXT1 protein is a type II transmembrane glycoprotein and comprises a Golgi-localized hetero-oligomeric complex that plays an integral part in heparan sulphate proteoglycan (HSPG) biosynthesis. Some research has shown that knockdown of EXT1 mRNA expression in osteochondromas was associated with intracellular accumulation of HSPGs in the Golgi apparatus. It has been shown that a lack of HSPGs on the cell surface affected growth signaling pathways in the growth plate, and possibly in osteochondromas [20] [36, 37]. In the growth plate, IHH requires interaction with HSPGs to diffuse through the extracellular matrix to its receptor [38]. These prior studies revealed that somatic mutations of EXT genes are extremely rare in non-hereditary osteochondroma. However, the observation that LOH and clonal rearrangement at 8q24 (EXT1 locus) are as frequent in non-hereditary osteochondromas as EXT1 gene mutations in patients with hereditary osteochondromas. This observation implied that EXT1 might be involved in the development of non-hereditary osteochondromas [35, 39]. Chen et al. [40] demonstrated that amplification of four genetic variations of EXT1 in four cases were identified. Thus, we detected that expression of EXT1 in both groups to determine the importance of the difference between condylar osteochondroma and condylar hyperplasia, and to preliminarily explore the mechanism of osteochondroma.

The positive rate of EXT1 expression in the condylar osteochondroma group was significantly higher than was found in condylar hyperplasia. EXT1 expression was concentrated on the cartilage layer. In addition, we can infer that over-expression of EXT1 may cause a disorder of endochondral ossification signaling cascades, leading to osteochondroma. Moreover, the negative expression of EXT1 in an all burn-out type IV specimen of condyle hyperplasia was shown to give rise to the relative relationship between EXT1 expression and cartilage formation in condylar osteochondroma.

## Conclusions

In summary, our semi-quantitative method for H&E and immunohistochemical staining showed that there was a thicker cartilage cap, a higher bone formation rate and higher PCNA positivity in condylar osteochondroma when compared to condylar hyperplasia, which indicated a higher proliferative activity of condylar osteochondroma. In addition, a higher EXT1 positive rate in condylar osteochondroma implied different biological characteristics in condylar osteochondroma when compared to condylar hyperplasia. These features might be useful in histopathologically distinguishing condylar hyperplasia and osteochondroma and in providing the basis for exploring the mechanism of condylar osteochondroma. However, its sensitivity and accuracy in clinical applications requires further study with a larger sampling set.

## Materials and methods

### Patients

This study was carried out at Shanghai Ninth People’s Hospital, Shanghai Jiaotong University School of Medicine. All patients were informed of the study purpose and gave consent. Eighteen cases with typical condylar osteochondroma, and 15 cases with typical condylar hyperplasia were treated in the Department of Oral and Craniomaxillofacial Surgery through 2005–2014. All cases were diagnosed based on the sequence including clinical features, representative computed tomography (CT) scan characteristics, single photon emission computed tomography (SPECT), and histopathological features (Fig. 1).

#### Diagnostic criteria

##### Unilateral condylar hyperplasia

(1) Clinical examination showed notable increases in ramous height and condyle neck height of the affected side that led to a rotated facial appearance and a canting occlusal plane. The prominence of the chin deviated to the contralateral side. In addition, temporomandibular disorder was detected in some cases.

(2) CT scans showed morphological enlargement of the condyle, and elongation and thickening of the condylar neck, presenting as an enlarged and smoothed condyle. Compared with the contralateral side, the uneven ossification was more significant and the trabeculae was larger with lower CT value detected. In addition, the characteristic cartilaginous cap was not seen.

(3) All cases with condylar hyperplasia were in the active phase, which was proven by follow-up visits for at least one year, with an SPECT value greater than 0.1.

(4) Post-surgery histopathological examiniation gave a diagnosis of Condylar hyperplasia.

##### Condylar osteochondroma

(1) Clinical examination showed facial asymmetry, hypomobility, deviation of the mouth opening and malocclusion. Occlusion plane canting was also measured. Some patients showed stable occlusion when assessing progress over a prolonged period of time. In addition, temporomandibular pain, noise and pre-auricular swelling was observed in some cases.

(2) CT scans showed cartilage cap covering the condylar surface and continuity of the cortex and trabeculae. The trabeculae was found to have an uneven ossification. The morphology of the condyle had clearly changed and was uneven in some cases, with lobulated surface or the formation of a pedunculated mass. The affected side of the TMJ joint surface of the temporal bone was reconstructed due to tumor compression, and the joint space was smaller than the contralateral side.

(3) The SPECT value of all cases with condylar ostechondroma exceeded a value of 0.1.

(4) Histopathological examination gave a diagnosis of condylar osteochondroma.

Furthermore, the surgical procedures by low condylectomy and orthognathic surgery spontaneously considering facial outcome and occlusion [6, 7]. Condylectomy included the lesion and value of decanting to correct the symmetry of the maxilla and mandible were performed in these 33 patients.

### Staining

The paraffin sections were derived from the resected condyle specimens mentioned above. The sections were dehydrated and embedded in paraffin following routine methods: the specimens were fixed in 4% paraformaldehyde for 24 h at 4 °C followed by decalcification with a decalcifying solution. The samples were then dehydrated in serially graded ethanol solutions, defatted in methanol and embedded in paraffin. The condyle sections were sagittally sectioned at a thickness of 5um, and deparaffinized in xylene, rehydrated in descending concentrations of alcohol, and stained with hematoxylin and eosin (H&E).

Immunohistochemistry was carried out by standard procedures. The sections had paraffin removed, which were then immersed in distilled water following routine methods. The sections were immersed in 1 mM pH 8.0 ethylene diamine tetraacetic acid (EDTA, Gibco, USA) solution and then heating in a water-bath for 25 min. Next, paraffin sections were rinsed three times for 3 min each in PBS pH 7.4 at room temperature. The sections were then incubated with the primary antibody, anti-EXT1 (1:150, Abcam, USA) and anti-PCNA (1:150, Santa Cruz Biotechnology, Inc., USA) at 4 °C overnight in a humidified chamber. After washing in PBS, the appropriate biotin-labeled secondary antibody was applied to the specimens. After rinsing three times for 3 min each in PBS pH 7.4, sections were exposed to DAB detection solution (DAKO, Denmark), following which the slides were treated in alcohol and xylene and then mounted with neutral balsam.

### Statistical analysis

Using the smallest scale of the 0.01 mm type, and the C1 eyepiece micrometer under × 200 magnification, we selected five fields of the thickest cartilage cap area of the H&E stained sections derived from both condylar hyperplasia and condylar osteochondroma, and then measured the thicknesses of the fibrous layer, the undifferentiated mesenchymal layer, the cartilage cell layer, and the depth of average infiltration. Then the numbers of cartilage islands were calculated respectively. We also took three images of bone tissue in the thickest cartilage cap area under × 50 magnification, and used image-j2x Image processing software to process the images and calculate the percentage of the osteogenic area.

The average number of PCNA positive cells was counted across five fields of view among the thickest cartilage-cap area in each section by two independent observers under a magnification of × 400 (Carl Zeiss Axioshop, German). And 200 cells and PCNA positive cells were counted on the microscope counting line to determine the positive rate in each field. Finally, the average positive rate was used as the PCNA proliferation index. Statistical analysis was performed using non-parametric Wilcoxon rank sum test (Mann-Whitney U test) of two independent samples was used to compare the differences between the indicators of the two diseases, and *P* < 0.05 was statistically significant by using the SPSS version 8.0 statistical software package (SPSS Inc., Chicago, IL).

EXT1 positive staining was located in cytoplasm, and the interpretation of EXT1 immunohistochemical results was based on Torlakovic EE’s method [41]: the definition of positive and negative in EXT1 immunohistochemical staining is bounded by 10% of positive cells (× 400 magnification). Thus, in this study 10% and more than 10% of the EXT1 staining was judged as positive. Uncolored or scattered staining fields, wherein the number of positive cells was less than 10% was judged to be negative. Statistical analysis was performed using Fisher exactly tested the comparison analysis using the SPSS version 8.0 statistical software package (SPSS Inc., Chicago, IL).

## Abbreviations

CT: Computerized tomography
DAB: Diaminobenzidine
EDTA: Ethylene diamine tetraacedic acid
EXT: Exostosin
H&E: Hematoxylin eosin
HS: Heparan sulfate
HSPGs: Heparan sulphate proteoglycans
IHC: Immunohistochemistry
MO: Multiple osteochondromas
MRI: Magnetic resonance imaging
PCNA: Proliferating cell nuclear antigen
SPECT: Single photon emission computed tomography
